# Optimized method for differential gene expression analysis in non-model species: Case of *Cedrela odorata* L.

**DOI:** 10.1016/j.mex.2023.102449

**Published:** 2023-10-18

**Authors:** Aragón-Magadán Marco Aurelio, Calvillo-Aguilar Francisco Fabián, Cruz-Cárdenas Carlos Iván, Guzmán Luis Felipe

**Affiliations:** Agricultural and Livestock Researches, National Genetic Resources Center, National Institute of Forestry, Jalisco, Mexico

**Keywords:** Bioinformatics, RNAseq, Transcriptomics, Linux, A pipeline for gene specific differential expression analysis in non-model species: case *Cedrela odorata* L

## Abstract

The following protocol introduces a targeted methodological approach of differential gene expression analysis, which is particularly beneficial in the context of non-model species. While we acknowledge that biological complexity often involves the interplay of multiple genes in any given biological response our method provides a strategy to streamline this complexity, enabling researchers to focus on a more manageable subset of genes of interest. In this context, red cedar transcriptome (*Cedrela odorata* L.) and known or hypothetical genes related to the response to herbivory were used as reference. The protocol key points are:

• Implementation of a transcriptome thinning process to eliminate redundant and non-coding sequences, optimizing the analysis and reducing processing time.
• Use of a custom gene database to identify and retain coding sequences with high precision.
• Focus on specific genes of interest, allowing a more targeted analysis for specific experimental conditions.

This approach holds particular value for pilot studies, research with limited resources, or when rapid identification and validation of candidate genes are needed in species without a reference genome.

## Graphical abstract

**Figure:**
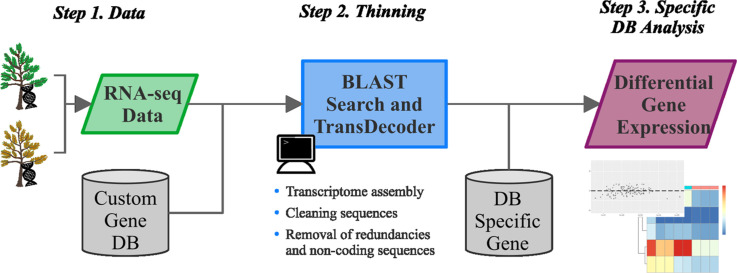

Specifications table

**Table:** 

Subject area:	Bioinformatics
More specific subject area:	Gene specific differential expression analysis
Name of your method:	A pipeline for gene specific differential expression analysis in non-model species: case *Cedrela odorata* L.
Name and reference of original method:	NA
Resource availability:	Docker; https://www.docker.com/ Anaconda; https://www.anaconda.com/ Ubuntu; https://ubuntu.com/ Fastp; https://github.com/OpenGene/fastp TrinityRNAseq; https://github.com/trinityrnaseq/trinityrnaseq/wiki Bowtie2 https://bowtie-bio.sourceforge.net/bowtie2/index.shtml BUSCO; https://busco.ezlab.org/ CD-HIT; https://sites.google.com/view/cd-hit Diamond; https://github.com/bbuchfink/ diamond Trans Decoder; https://github.com/TransDecoder/ TransDecoderLAST+;https://blast.ncbi.nlm.nih.gov/doc/blasthelp/downloadblastdata.html Seqkit; https://bioinf.shenwei.me/seqkit/

## Method details

Differential gene expression analysis constitutes the primary application of data derived from RNAseq sequencing. This methodological approach facilitates the identification of genes or transcripts that display differential expression in samples with significant contrasts. This process is essential for understanding gene regulation and biological responses to various experimental or pathological conditions [1].

RNA-seq data frequently encapsulates a multifaceted landscape of gene expression, influenced by a myriad of factors such as biological and technical noise [2,3]. This complexity is further amplified in non-model species, where comprehensive transcriptomic datasets are often lacking [2]. To address these challenges, our method concentrates on a curated panel of genes with known or hypothesized functions. It is crucial to note that this targeted approach does not imply the irrelevance of genes outside this curated panel. Rather, the method is designed to yield a dataset that is both manageable and interpretable, thereby facilitating the investigation of specific research questions.

## RNAseq data

Eleven paired 75 bp RNAseq libraries corresponding to red cedar *(Cedrela odorata* L.) were sequenced at the DNA and Genomics Laboratory of the National Center for Genetic Resources of INIFAP. The experimental conditions included six individuals showing signs of attack from the shoot borer (*Hypsipyla grandella*) and five healthy plants.

## Operating system and software

The Linux OS, Ubuntu version 22.10, was used, installed on an Intel Xeon E510 computer with 120 GB of RAM and 64 CPUs. Anaconda 3.11 and Docker 23.0.3 were employed for software package installation.

## Library cleaning

(1) Library cleaning with fastp [4].

> fastp -i file1_R1.fastq -I file1_R2.fastq -o clean_file1_R1.fastq -O clean_file1_R2.fastq -l 20 -q 20

The ``-l'' flag instructs fastp that the minimum read length is 20 bp, and the ``-q'' specifies that the minimum quality score is 20.

## Transcriptome assembly and quality assessment

For the purpose of conducting *de novo* transcriptome assembly, the selection of a suitable software tool is at the discretion of the researcher. However, in this protocol, we provide instructions for conducting it with Trinity RNAseq [5]. It is recommended to use the Docker version provided by the authors.

(1) De novo transcriptome assembly using all RNAseq libraries:
  > cat clean*_R1.fastq > all_R1.fastq
  > cat clean*_R2.fastq > all_R2.fastq
  > Trinity –seqType fq –left all_R1.fastq –right all_R2.fastq –full_cleanup –output Trinity_reference
(2) Reads support analysis with Bowtie2 [6].
  a Index creation for Bowtie2:
  > bowtie2-build Trinity_reference.fasta out_bowtie2_index
  b Alignment of RNAseq libraries with Bowtie2.
  > bowtie2 -x out_bowtie2_index −1 all_R1.fastq −2 all_R2.fastq –sensitive –local -S out.sam 2>&1 | tee result_bowtie2.log

The results from Bowtie2 are displayed upon completion of the program's execution. By using the option “2>&1 | tee result_bowtie2.log”, the results are also saved in a file named “result_bowtie2.log”, allowing for future reference if necessary.

If the read alignment exceeds 70%, it is considered that an appropriate assembly was performed. However, if it is lower, it might indicate poor quality in the assembly, and it is suggested to consider the possibility of repeating the process or discarding the data, as appropriate [7].

(3) General assembly quality assessment with BUSCO [8].
  > busco -m transcriptome -l embryophyta -i Trinity_reference.fasta -o output_busco

The flag ``-m transcriptome'' indicated that BUSCO performed the gene search in transcriptome mode, while the “-l embryophyta” flag corresponds to the lineage of the analyzed organism. This step is essential in de novo assemblies, as it allows for the search of functional genes. Homology values above 80% indicate good assembly quality [7,8].

## Removal of redundancies and non-coding sequences from the transcriptome

Transcriptomes encompass a substantial quantity of redundant and non-coding sequences, thereby impeding downstream analyses by demanding greater processing power and execution time [9,10]. Therefore, it is necessary to remove these sequences. This process is known as transcriptome thinning [11].

(1) Redundancy removal from the transcriptome using CD-HIT [12].

> cd-hit-est -c 0.9 -i Trinity_reference.fasta -o cd-hit-reference.fasta

The flag ``-c 0.9'' specifies that the similarity value for CD-HIT was set at 90%. This means that the program grouped sequences that share a 90% similarity with each other and selects one representative sequence per group, thus eliminating redundancies.

(2) Removal of non-coding sequences using diamond [13] and TransDecoder.

a Users can choose the genomic database of their preference. However, for the purposes of this protocol, a custom protein database corresponding to embryophytes was constructed, downloaded from UNIPROT [14], with a total of 8101,067 sequences.
b Diamond database.

  > diamond makedb --in uniprot.fasta -d diamond_db

c Translation of the redundancy-free transcriptome to proteins.

  > Transdecoder.LongOrfs -t cd-hit-reference.fasta

d BLASTP with diamond.

> diamond blastp –fast –max-target-seqs 5 –outfmt 6 –query transdecoder.pep –db diamond_db –outfmt 6 –out blastp.fmt6

The flag ``–max-target-seqs 5'' indicates that the top 5 alignments are reported for query sequences. "–outfmt 6″ means that the diamond result is saved in tabular BLAST format. It's important to note that TransDecoder generates files with various extensions, so to perform the BLASTP, it is necessary to use the file with the ``.pep'' extension.

The results from diamond are essential for TransDecoder to more accurately predict coding sequences. The decision to use this program was based on its speed and efficiency in handling large volumes of data [12].

e Predicting Coding Sequences with TransDecoder.

> TransDecoder.Predict -t cd-hit-reference.fasta –retain_blastp_hits blastp.fmt6.

In the context of the TransDecoder framework, the command-line option "–retain_blastp_hits blastp.fmt6″ is utilized to incorporate the results obtained from the diamond BLASTP algorithm. This action serves to retain sequences that have exhibited significant matches in the BLASTP analysis. This procedure improves the precision of coding sequence predictions. At the conclusion of this process, the resultant nucleotide coding sequences were preserved within the ".cds" file, while the corresponding amino acid protein sequences were stored in the ".pep" file.

## Reference transcriptome

Once the thinning process is completed, a transcriptome will be obtained that can be used as a reference for differential expression analysis. That is, the researcher may choose to conclude the protocol at the previous point. However, if the primary objective is to conduct an analysis focused on a specific group of genes, it is recommended to proceed with the following steps.

## Reference transcriptome for specific genes

(1) The researcher can choose to download the group of genes of interest from any of the available genomic databases. However, for the purposes of this protocol, searches for embryophyte protein sequences containing the keyword "herbivore" in UNIPROT were conducted, and a custom database was generated using BLAST+ [15].

a BLAST gene database.

> makeblastdb -dbtype prot -in uniprot_herbivore.fasta -out blast_herbivore.

The flag ``-dbtype prot'' specifies that the sequences used correspond to proteins. If the data are nucleotide sequences, the type should be changed to ``-dbtype nucl''.

b Searching for homologous genes by BLAST.

> blastp –max_target_seqs 5 -db blast_herbivore –query transdecoder-cd-hit-reference.pep –outfmt ‘6 qseqid sseqid salltitles pident qcovs evalue’ -out blastp_herbivore.fmt6.

The “-outfmt” flag contains several fields that will produce an output in BLAST tabular format with columns in the following order: Query ID, Subject ID, All subjects titles, Identity percent, Query coverage per subject, and Evalue. This information will be very helpful for subsequent information extraction.

TIP: It is recommended to save the BLAST result in ASN.1 archive format (-outfmt 11) and then reformat with blast_formatter to BLAST tabular (-outfmt 6). The ASN.1 format contains all the metadata information related to the BLAST results, which can be useful for future queries.

c Extraction of homologous sequences from the reference transcriptome using seqkit [16].

> cat blastp_herbivore.fmt6 | awk ‘{ if ($4 >=30 && $5>=50){print}}’ | cut -f 1 | sort | uniq > transcripts_list.txt

> seqkit grep -f transcripts_list.txt transdecoder-cd-hit-reference.cds > transcripts_hervibore.fasta.

In Linux, the pipe symbol (|) is used to sequentially execute a series of commands applied to the same dataset. In the first line of code, ``cat'' opens the “blastp_herbivore.fmt6” file, which is the result of the BLAST. Then, “awk” applies a filter to the data to print only those with an identity percentage value (column 4, $4) greater than or equal to 30% and a coverage percentage (column 5, $5) greater than or equal to 50%. It is important to note that in protein alignments, sequences are considered homologous if the similarity and coverage percentage exceeds 30% and 50% respectively [15]. Next, the ``cut'' command extracts column 1 (-f 1) which contains the query IDs, then ``sort'' and ``uniq'' sort alphabetically and remove duplicate IDs. The result of this entire process was saved in the ``transcripts_list.txt'' file.

In the second line of code, the ``grep'' option indicates to seqkit to use the sequence search function by ID and using ``-f'' specifies that the IDs are found within the ``transcripts_list.txt'' file. It's important to highlight that the search for the transcripts must be carried out in the TransDecoder results specifically within the file with the ``.cds'' extension.

## Conclusion

Two differential gene analyses were conducted on 11 RNAseq libraries from *Cedrela odorata* L. The first was executed conventionally using the entire transcriptome. The second was conducted following the protocol described in this paper. In both cases, transcript quantification was carried out with Salmon [17], and the differential expression analysis was performed in R using the DESeq2 package [18].

**Fig. 1:**
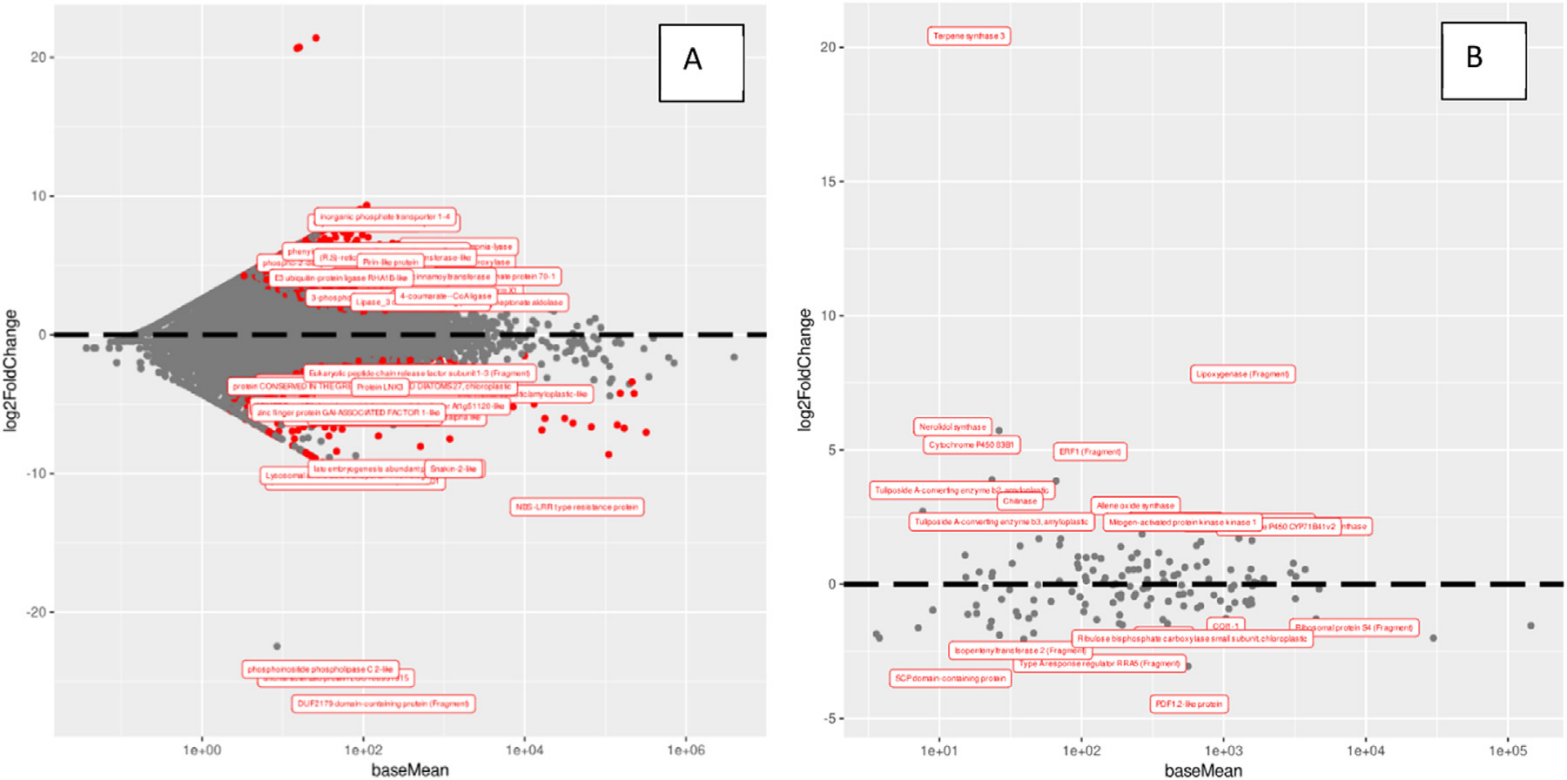
(A) Whole transcriptome analysis, 819 differentially expressed genes. (B) Analysis targeting genes related to defense against herbivory, 22 differentially expressed genes.

**Fig. 2:**
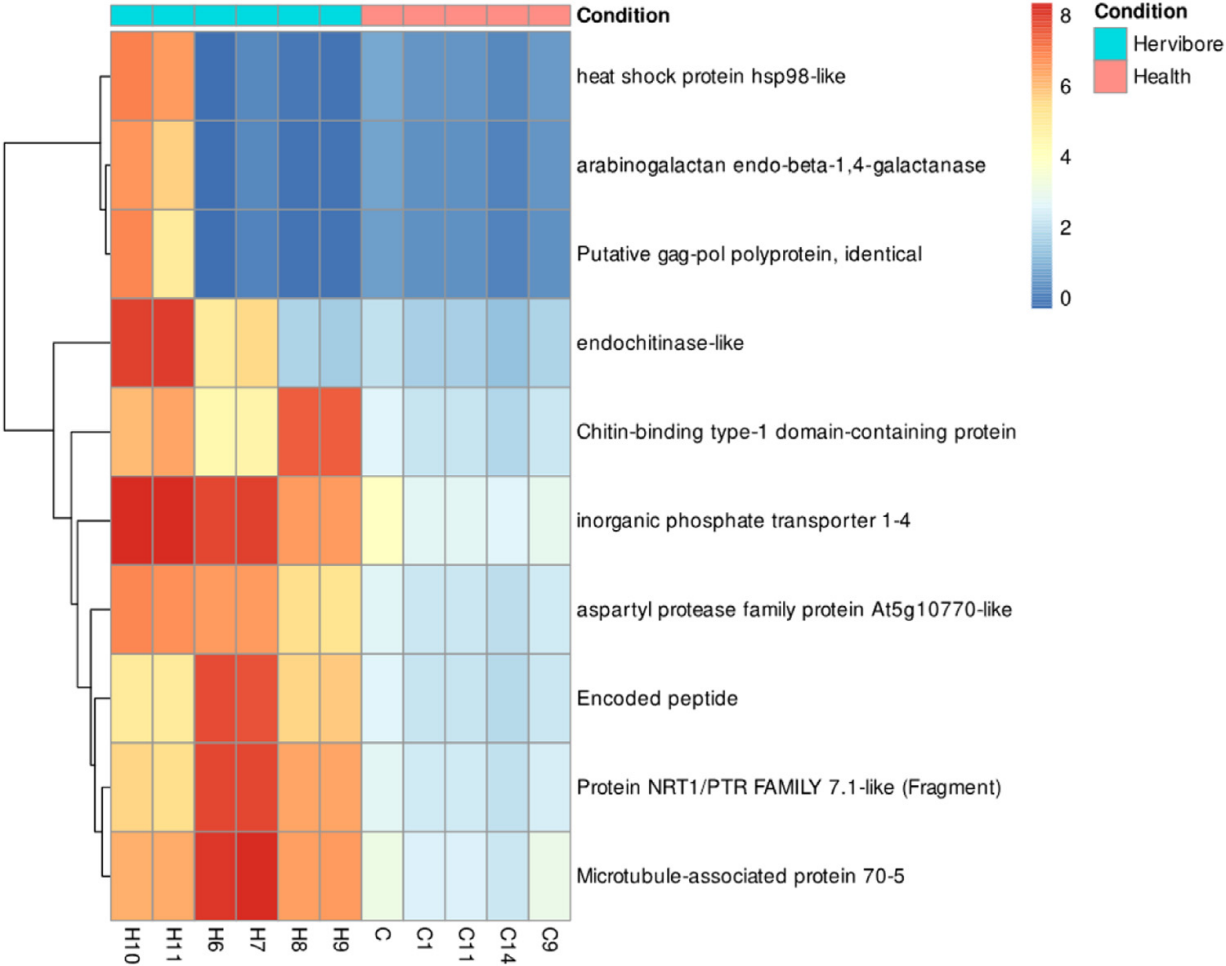
Heatmap of the complete transcriptome analysis. The top ten differentially expressed genes are not reported as related to defense against herbivores.

Our findings demonstrate a notable contrast between a whole-transcriptome approach and the targeted methodology proposed in this study for differential gene expression analysis. Utilizing a comprehensive transcriptomic analysis, we identified a substantial set of 819 differentially expressed genes (Fig. 1). It is important to note that the relevance of these genes to herbivore attacks is not immediately discernible, as illustrated by the heat maps of the top ten differentially expressed genes (Fig. 2) [19], [20], [21], [22], [23]. In contrast, our targeted approach yielded a more focused set of 22 differentially expressed genes (Fig. 1) [19], [20], [21], [22], [23]. This is not to suggest that genes identified through whole-transcriptome analysis are inconsequential. Rather, it highlights the efficacy of a targeted approach in generating a dataset that is both interpretable and tailored to address specific research questions.

Our findings indicate that while whole-transcriptome analysis offers a comprehensive perspective on gene expression changes, it may also yield a complex dataset influenced by various factors such as biological variability and noise [1,3,10]. On the other hand, a targeted approach, such as the one presented in this study, provides a more focused dataset that is easier to interpret for specific research questions. It is important to note that the targeted approach is not intended to dismiss the potential relevance of other genes but rather to offer a more streamlined method for investigating specific biological phenomena.

## Ethics statements

The data used for this protocol were provided by the DNA and Genomics Laboratory of the National Center for Genetic Resources of INIFAP.

## CRediT authorship contribution statement

**Aragón-Magadán Marco Aurelio:** Conceptualization, Formal analysis, Investigation, Project administration, Software, Supervision, Writing – original draft, Writing – review & editing. **Calvillo-Aguilar Francisco Fabián:** Conceptualization, Formal analysis, Investigation, Project administration, Software, Supervision, Writing – original draft, Writing – review & editing. **Cruz-Cárdenas Carlos Iván:** Conceptualization, Formal analysis, Investigation, Project administration, Software, Supervision, Writing – original draft, Writing – review & editing. **Guzmán Luis Felipe:** Conceptualization, Formal analysis, Investigation, Project administration, Software, Supervision, Writing – original draft, Writing – review & editing.

## Data Availability

Data will be made available on request.
